# Physical qualities and body composition predictors of running performance in national level women’s official soccer matches

**DOI:** 10.5114/biolsport.2023.118026

**Published:** 2022-09-15

**Authors:** Eero H.J. Savolainen, Tomi Vänttinen, Johanna K. Ihalainen, Simon Walker

**Affiliations:** 1 Faculty of Sport and Health Sciences, University of Jyväskylä, Finland; 2 KIHU – Research Institute for Olympic Sports, Jyväskylä, Finland

**Keywords:** Football, High intensity running, Anthropometrics, Female, Match demands

## Abstract

The purpose of the study was to (1) determine match running performance, anthropometry and various physical qualities of national level women soccer players and (2) identify key physical qualities and anthropometric predictors of match running profile during a competitive season. Twenty-five national level Finnish soccer players participated in the study. Players performed countermovement jump, loaded squat jumps, 30-meter sprint, maximum isokinetic knee flexor and extensor contractions, an incremental treadmill test and underwent body composition assessment in the lab. Match running performance was analyzed from 115 match observations during competitive league matches over 11 weeks after the laboratory tests. Pearson’s correlation was used to determine bivariate relationships between match running variables and physical qualities and anthropometric variables. Identified significant bivariate relationships were then entered into multiple regression analyses to identify the best predictors of match running performance. Physical qualities and anthropometric variables predicted 65% of very high-intensity (VHIR) (> 19 km/h) and 63% of high-intensity (HIR) (13–19 km/h) running distances covered during matches, but only 22% of low-intensity (LIR) and 43% of total distances. Body fat percentage and high-speed knee flexor concentric strength were the most important predictors to VHIR and HIR while aerobic capacity-related variables were most important predictors to LIR and total distance. Physical qualities and anthropometry can predict a large portion of players’ VHIR and HIR performance during matches in women’s national level soccer. To increase player’s VHIR and HIR distance, coaches could aim to develop players’ high-speed (especially knee flexor concentric) strength and optimize player’s body composition.

## INTRODUCTION

In the 2019 World Cup, elite (outfield) women soccer players covered an average 9–11 km during the match, in which they averaged 349–591 meters at speeds of 19–23 km/h and 111–255 meters at speeds of > 23 km/h [1]. It is known that match running demands increase in-line with competition level [2, 3]. From match running variables, high-intensity running (HIR) should be emphasized because straight sprinting is the most frequent action in goal situations, at least in men’s professional soccer [4]. Even though a player’s running performance in soccer is a complex phenomenon, differing depending on multiple factors such as player’s position [5], aerobic capacity [6, 7], opposition standard [8], field surface [9], match outcome [9, 10], heat and altitude [10], it is important to be able to recognize which physical qualities and anthropometric variables predict match running performance. The identified qualities could then be improved to allow player development to fulfill match running demands of a higher level.

Elite women soccer players have higher physical qualities compared to lower-level counterparts [11, 12]. Previous studies have shown that, on average, elite women soccer players’ maximal oxygen uptake (VO_2_max) varies from 50 to 55 ml/kg/min [7, 12, 13, 14], countermovement jump height from 30 to 36 cm [11, 13, 15] and time in a 30-meter sprint test from 4.35 to 4.55 seconds [11, 15]. The relationship between match running performance and physical qualities is extensively documented in elite men and in male youth players, but very few studies have examined women players [16]. Correlation coefficients between total and HIR distances covered during the match and performance in different variations of yoyo--tests have varied considerably in women soccer players (r = 0.12–0.76) [6, 7, 17, 18]. Although previous studies have found strong correlations between VO_2_max and HIR (r = 0.76–0.83) [7, 19], there are disparities in coefficients between VO_2_max and total distance covered during the match (r = 0.20–0.76 [7, 19]). McCormack et al. [19] examined predictors of HIR capacity in collegiate women players and found that VO_2_max, dominant leg vastus lateralis thickness and pennation angle were the strongest predictors of HIR distance.

Associations between match running performance and strength, speed or power qualities in women soccer players are not widely studied, and findings from these few studies have been partly conflicting [17, 18]. It seems that the number of high-intensity accelerations is the in-match variable that is most related to strength (r = 0.26–0.49), power (r = 0.24–0.76) and speed qualities (r = -0.34 to -0.77) [17, 18]. In terms of high-speed/sprint distances covered during matches, Goncalves et al. [17] found weak-to-strong associations with power (r = 0.11–0.56) and speed qualities (r = -0.11 to -0.70), while Villaseca-Vicuna et al. [18] did not find any significant correlations between high-speed running distance and strength, speed or power qualities. Further, the only association between strength, speed and power qualities and total distance reported in women players is a surprising significant negative correlation (r = -0.40) between back squat 1RM and total distance [18]. This conflicts a finding in elite men players that greater muscle strength helped to maintain running distance throughout the match [20]. Demonstrating the confusion in the literature whether such physical qualities can predict match running performance at present.

Previous studies regarding women soccer players’ anthropometry have shown that elite women are ~167–172 cm tall, weigh ~60–64 kg and have a fat percentage of ~20–22% [14, 15, 21], but there are no studies that would have examined associations between women players’ anthropometry and match running performance. Some previous studies in elite men have shown sporadic associations between anthropometry and match running performance [22, 23]. However, one recent study found systematic associations between body fat percentage and HIR (r = -0.38) as well as sprint running (r = -0.57) distances in professional men players [24]. Thus, associations between these variables should be tested and confirmed in women soccer players to inform coaching practices.

Even if women’s soccer has grown in popularity a lot during recent years [1], there is a lack of knowledge regarding how anthropometry and various physical qualities as well as their inter-relationships, influence match running performance in national level women’s soccer. Therefore, coaching practice and targeted player development cannot be optimized. Hence, the purpose of the study is to (1) identify physical qualities, anthropometry and match running profiles of national level women soccer players, and (2) determine which physical and anthropometric qualities predict match running performance during a competitive season.

## MATERIALS AND METHODS

### Participants

Thirty-six national level [25] women soccer players from a single club in Finland volunteered for the study. Twenty-five players fulfilled the inclusion criteria; 1) outfield playing position, 2) performed fitness tests before the start of the match season, and 3) played at least 75 minutes in two matches during an 11-week period after the tests, and were included in data analyses. Players were from two teams: the 1^st^ team (n = 14, 6 defenders, 5 midfielders and 3 attackers, age 21.7 ± 2.5 years) playing in the highest national league, and the U18-team (n = 11, 4 defenders, 4 midfielders and 3 attackers, age 16.9 ± 0.9 years) playing in the highest U18-national league. All participants provided written informed consent prior to testing, the study was conducted according to the Declaration of Helsinki (2013) and was approved by the ethics committee of the University of Jyväskylä (5U/2019).

### Experimental overview

This study was a prospective, observational study performed during the 2020 competitive season. Physical quality and body composition tests were performed 1–2 weeks before the start of the league season and match data was collected during an 11-week period after the tests, when teams played against all other teams in their league once. The 1^st^ team played 9 matches during the data collection period (2 wins, 1 draw and 6 losses) and U18-team played 10 matches (5 wins, 3 draws and 2 losses). Two matches (both wins) from the U18-team were excluded from analyses because of hardware related technical problems. Both teams played in a 4-4-2 formation. Players performed all fitness tests during a single session in the laboratory, which took approximately two hours. The tests began with a standardized warm-up (5-min cycling, 10 reps of squats, lunges and hip thrusts and dynamic mobility exercises for lower limbs) followed by countermovement jump and loaded squat jump tests. Thereafter, players performed a 30-meter sprint test and isokinetic strength tests. The session ended with an incremental running test on a treadmill. Players used running shoes in all laboratory tests. Body composition measurement was performed on a separate morning after a 12 h overnight fast.

### Measurements

Match activity data was collected and analysed from competitive league matches using polar team pro player tracking system (Polar Electro Oy, Kempele, Finland) with GPS sampling at 10 Hz. Ten Hz GPS devices have been shown to be valid and reliable across linear and team sport simulated running [26]. Good-to-moderate reliability (< 5% CV) and validity for total distance, linear running and team sport simulation circuit have been shown for the same Polar team pro system used in the present study [27]. The following variables were used to represent match activity: total distance [m] covered during the match, distance covered [m] in speed zones 1–5: zone 1 (< 7 km/h), zone 2 (7–13 km/h), zone 3 (13–19 km/h), zone 4 (19–23 km/h and zone 5 (> 23 km/h), which were similar to thresholds used for the FIFA Women’s World Cup 2019 [1]. For correlation and regression analyses, distance covered in zones 1 and 2 were combined into one variable to describe distance covered in low-intensity running (LIR, 0–13 km/h) and zones 4 and 5 were combined into one variable to describe very high-intensity running (VHIR, > 19 km/h). Zone 3 was used to describe high-intensity running (HIR, 13–19 km/h).

Overall, 115 (65 from the 1^st^ team and 50 from U18-team players) individual match activity observations were used in this study and each player’s individual match activity profile was calculated from 4.6 ± 2.3 matches (1^st^ team players 4.6 ± 2.5 and U18-team players 4.6 ± 2.1, range 2–8). Players’ match activity data was accepted to the analyses if the player played more than 75 minutes in at least two matches. Match activity data was standardized to 90 min values, when added time after first and second halves were excluded. Most previous studies have included match data only if a player has played full match (90 minutes) [3, 7, 28] but in some studies analyses have even been performed from as little as 15 [29] or 20 [30] minutes of match play. In the present study, 75 minutes was selected as the acceptance threshold, because the introduction of a 5-substitute rule due to COVID-19 led to more frequent substitutions and a lower number of players who played until full-time in the matches. One [19] or two [6, 28] matches per player have been used in previous similar studies to investigate match performance analysis.

In the lab, countermovement jump height was measured using an infrared mat (Spintest, Estonia). Players performed two warm-up trials and four test trials. The highest jump was used in final analysis. Rest intervals between trials were one minute. A loaded squat jump test modified from Samozino et al’s [31] method was used to evaluate players’ force, velocity and power output in a ballistic movement. In the present study, players performed jumps using four different loads, which varied from 0–55% of body mass and loads were performed in increasing order. Evaluations of force, velocity and power developed by the lower-limb extensor muscles during squat jumps were calculated by the computations provided by Samozino et al. [31]. The same equipment was used as in countermovement jump test.

For the 30-meter sprint test, players started 50 cm behind single beam photocell gates (Newtest Oy, Finland), which were 30 cm from the ground. Players performed two warm-up trials and three test trials, and the best time was used in analyses. Rest intervals between trials were two minutes.

Peak knee extensor and flexor force in both concentric and eccentric muscle actions were measured using two different speeds: 60 and 180 degrees per second. The range of motion of the dynamometer (Custom built in University of Jyväskylä) was 78 degrees (knee angle 90–168 degrees, 180 = fully extended). Players performed four trials in each condition separated by 20-second rest interval between trials [32]. Subjects always performed knee extensor followed by knee flexor trials and a speed of 60 degrees per second followed by 180 degrees per second. The order of muscle action (concentric or eccentric) was randomized. The highest peak torque of each condition adjusted to the subject’s body mass was used in final analyses. The test was performed by the right leg only. Based on a meta-analysis between-limbs, muscle strength measured by maximal isokinetic dynamometry demonstrates symmetry across ages, genders, and levels of play in soccer players [33].

Maximal oxygen uptake was measured by an incremental treadmill (Telineyhtymä Oy, Kotka, Finland) test (starting speed 7 km/h, 1 km/h increase every 3^rd^ minute) following methods from Vesterinen et al. [34], except that the highest running speed (sMax) of the test was defined as the highest completed speed level. Oxygen consumption was measured breath-by-breath (OxyconPro, Jaeger, Hochberg, Germany) and heart rate was monitored continuously (Polar V800, Polar, Kempele, Finland). The determination of lactate thresholds was based on the first rise and change in inclination of the blood lactate curve during the test [34].

Body composition was measured following an overnight fast using dual-energy X-ray absorptiometry (DXA) and Encore software (version 9.3, LUNAR Prodigy Advance; GE Medical Systems, Chicago, IL) as described previously [35].

### Statistical analyses

Statistical analyses were conducted using SPSS Statistics 24 (IBM, Armonk, NY). Results are reported as means ± standard deviation (SD). Data normality was assessed using the Shapiro-Wilk test and all variables were normally distributed (distance covered in zone 5 after logarithmic transformation). Between-group differences in physical quality, anthropometric and match running variables were analyzed using independent t-test. For pairwise comparisons, effect sizes were calculated by using Hedges’ *g*. Effect sizes were classified using the following criteria: 0.2–0.5 small, 0.5–0.8 medium, > 0.8 large. As there were only minor differences between groups in physical qualities, anthropometric and match running variables, groups were combined to form a larger sample to correlation and regression analysis, decreasing the possibility of statistical error through small sample size.

Pearson’s product moment correlation was used to assess correlations between match activity variables, physical qualities, and body composition variables. Correlation magnitudes were classified using the following criteria: < 0.3 weak; 0.3–0.7 moderate, > 0.7 strong. To identify the variables most strongly predictive of match running performance, multiple regression analyses were conducted by automatic linear modelling (forward stepwise method). Those parameters significantly related with a selected match running variable in the Pearson’s correlations were introduced into the regression models. Significance level was set at p < 0.05.

## RESULTS

*Group differences*. Table 1 shows the descriptive characteristics for the 1^st^ team and U18-team players. The only statistically significant difference between the two teams was found in knee extensor peak torque in concentric action with a speed of 180 degrees per second.

**TABLE 1:** Descriptive characteristics for the 1st team and U18 team players, and average values (mean + SD).

**A**	**Age (years)**	**Height (cm)**	**Body mass (kg)**	**Body mass index**	**Fat percen-tage (%)**	**Lean mass (kg)**	**30-m sprint (s)**	**CMJ (cm)**	
Average	19.6 (3.1)	169.1 (4.9)	64.5 (9.4)	22.4 (2.8)	24.3 (5.1)	45.6 (5.5)	4.67 (0.16)	31.2 (3.3)	
First team	21.7 (2.5)	168.0 (5.3)	64.2 (10.2)	22.6 (2.7)	23.3 (5.2)	46.0 (6.4)	4.63 (0.18)	32.3 (3.4)	
U18 team	16.9 (0.9)	170.0 (3.9)	64.8 (8.6)	22.2 (3.1)	25.8 (5.0)	44.8 (6.4)	4.74 (0.12)	30.0 (2.8)	
p-value		0.158	0.882	0.759	0.249	0.570	0.118	0.080	
Hedges’ g		-0.566	-0.060	0.132	-0.486	0.215	-0.633	0.714	

**B**	**VO_2_max (ml/kg/min)**	**sMax (km/h)**	**LT2 VO_2_ (ml/kg/min)**	**LT2 speed (km/h)**	**LT1 VO_2_ (ml/kg/min)**	**LT1 speed (km/h)**	**F0 (N/kg)**	**V0 (m/s)**	**P_max_ (W/kg)**
Average	45.8 (3.3)	14.9 (1.1)	40.6 (3.6)	11.8 (0.9)	33.8 (2.8)	9.4 (0.8)	30.8 (3.5)	2.28 (0.26)	17.5 (1.6)
First team	46.0 (2.4)	15.2 (1.1)	40.5 (2.8)	11.9 (0.8)	33.6 (2.5)	9.5 (0.7)	31.5 (3.3)	2.23 (0.26)	17.7 (1.5)
U18 team	45.6 (4.2)	14.5 (1.1)	40.9 (4.6)	11.8 (1.1)	34.1 (3.4)	9.4 (1.1)	30.0 (3.6)	2.35 (0.24)	17.4 (1.7)
p-value	0.825	0.140	0.767	0.751	0.666	0.875	0.299	0.267	0.707
Hedges’ g	0.087	0.596	-0.117	0.125	-0.170	0.062	0.414	-0.443	0.148

**C**	**Econ60 (Nm/kg)**	**Eecc60 (Nm/kg)**	**Econ180 (Nm/kg)**	**Eecc180 (Nm/kg)**	**Fcon60 (Nm/kg)**	**Fecc60 (Nm/kg)**	**Fcon180 (Nm/kg)**	**Fecc180 (Nm/kg)**	
Average	2.7 (0.5)	4.3 (0.7)	1.9 (0.4)	3.9 (0.8)	1.3 (0.2)	1.8 (0.4)	1.0 (0.2)	1.8 (0.3)	
First team	2.9 (0.3)	4.5 (0.8)	2.1 (0.3) *	4.2 (0.9)	1.2 (0.2)	1.7 (0.3)	1.0 (0.2)	1.7 (0.3)	
U18 team	2.5 (0.6)	4.0 (0.5)	1.5 (0.3) *	3.6 (0.4)	1.4 (0.3)	1.8 (0.4)	1.1 (0.3)	1.9 (0.3)	
p-value	0.067	0.080	< 0.001	0.054	0.257	0.606	0.370	0.277	
Hedges’ g	0.868	0.727	1.790	0.772	-0.505	-0.213	-0.399	-0.432	

**D**	**Totaldistance (m)**	**Zone 1 distance (m) (0–7 km/h)**	**Zone 2 distance (m) (7–13 km/h)**	**Zone 3 distance (m) (13–19 km/h)**	**Zone 4 distance (m) (19–23 km/h)**	**Zone 5 distance (m) (> 23 km/h)**	**LIR distance (m) (< 13 km/h)**	**HIR distance (m) (13–19 km/h)**	**VHIR distance (m) (> 19 km/h)**
Average	9203 (882)	3468 (291)	3840 (620)	1600 (405)	238 (109)	56 (60)	7308 (485)	1600 (405)	295 (161)
First team	9205 (871)	3457 (298)	3811 (528)	1623 (436)	248 (119)	66 (72)	7268 (489)	1623 (436)	315 (184)
U18 team	9200 (938)	3481 (295)	3878 (746)	1571 (380)	226 (99)	45 (40)	7359 (499)	1571 (380)	271 (131)
p-value	0.989	0.843	0.794	0.758	0.620	0.385	0.754	0.652	0.492
Hedges’ g	0.005	-0.078	-0.103	0.122	0.196	0.345	-0.179	0.122	0.262

A = anthropometry, 30-meter sprint speed and countermovement jump height (CMJ), B = Maximal oxygen consumption (VO_2_max) and maximal speed during VO_2_max test (sMax), speeds at lactate thresholds 1 (LT1 speed) and 2 (LT2 speed). Oxygen consumption at lactate thresholds 1 (LT1 VO_2_), 2 (LT2 VO_2_) and force (F0), velocity (V0) and power (Pmax) outputs from loaded squat jumps. C = Body mass adjusted peak torques of knee extensors in concentric action with 60 degrees (Econ60) and 180 degrees (Econ180) per second and in eccentric action with 60 degrees (Eecc60) and 180 degrees (Eecc180) per second. Body mass adjusted peak torques of knee flexors in concentric action with 60 degrees (Fcon60) and 180 degrees (Fcon180) per second and in eccentric action with 60 degrees (Fecc60) and 180 degrees (Fecc180) per second. D = Distance covered in different speed zones in matches and combinations of speed zones: low-intensity running (LIR), high-intensity running (HIR) and very-high-intensity running (VHIR) used in later analysis.

* = Statistically significant difference between groups, p < 0.05.

*Bivariate relationships*. Table 2 shows correlation coefficients between match running and physical and anthropometrical variables. Several statistically significant (positive and negative) moderate-to-strong correlations were observed between specific variables.

**TABLE 2:** Correlation coefficients between match running and physical and anthropometrical variables in both groups together.

	Total distance (m)	LIR distance (m) (0–13 km/h)	HIR distance (m) (13–19 km/h)	VHIR distance (m) (> 19 km/h)
Height (cm)	-0.13	-0.04	-0.10	-0.31
Body mass (kg)	-0.42*	-0.21	-0.43*	-0.55*
Body mass index	-0.41	-0.21	-0.43*	-0.49*
Fat percentage (%)	-0.56*	-0.28	-0.59*	-0.73*
Lean mass (kg)	-0.18	-0.07	-0.18	-0.30
F0 (N/kg)	0.47*	0.39	0.42*	0.36
V0 (m/s)	-0.20	-0.15	-0.20	-0.15
Pmax (W/kg)	0.37	0.25	0.28	0.55*
30-m sprint speed (s)	-0.23	-0.14	-0.16	-0.44*
CMJ (cm)	0.16	0.11	0.05	0.39
VO_2_max (ml/kg/min)	0.57*	0.46*	0.54*	0.41*
Max speed (km/h) in VO_2_max test	0.21	0.27	0.21	-0.21
LT2 VO_2_ (ml/kg/min)	0.56*	0.41*	0.52*	0.51*
LT2 speed (km/h)	0.56*	0.50*	0.55*	0.19
LT1 VO_2_ (ml/kg/min)	0.29	0.13	0.27	0.48*
LT1 speed (km/h)	0.53*	0.45*	0.52*	0.22
Econ60 (Nm/kg)	0.23	0.22	0.18	0.16
Eecc60 (Nm/kg)	0.07	0.02	0.06	0.24
Econ180 (Nm/kg)	0.35	0.17	0.31	0.59*
Eecc180 (Nm/kg)	0.10	0.17	-0.01	0.11
Fcon60 (Nm/kg)	0.29	0.22	0.28	0.25
Fecc60 (Nm/kg)	-0.20	-0.23	-0.13	-0.09
Fcon180 (Nm/kg)	0.45*	0.23	0.52*	0.43*
Fecc180 (Nm/kg)	-0.07	-0.01	-0.07	-0.18

Distances covered in low-intensity running (LIR), high-intensity running (HIR) and very-high-intensity running (VHIR). Force (F0), velocity (V0) and power (P_max_) outputs from loaded squat jumps. Countermovement jump height (CMJ), Maximal oxygen consumption (VO_2_max) and maximal speed during VO_2_max test (sMax), speeds at lactate thresholds 1 (LT1 speed) and 2 (LT2 speed). Oxygen consumption lactate thresholds 1 (LT1 Vo2) and 2 (LT2 Vo2). Peak torques of knee extensors in concentric action with 60 degrees (Econ60) and 180 degrees (Econ180) per second and in eccentric action with 60 degrees (Eecc60) and 180 degrees (Eecc180) per second. Peak torques of knee flexors in concentric action with 60 degrees (Fcon60) and 180 degrees (Fcon180) per second and in eccentric action with 60 degrees (Fecc60) and 180 degrees (Fecc180) per second.

* = Statistically significant correlation between selected anthropometry or physical variable and running distance in selected intensity, p < 0.05.

*Match running performance predictors*. Table 3 shows linear regression models that predict total distance and distance covered in different speed zones in matches. Regression equations were:

**TABLE 3:** Regression models to predict match running variables.

Dependent variable	Adjusted R^2^	Predictors	B (95%CI)	Standard error	Importance	Significance
Total Distance (m)	0.426					
		Constant	1106.58 (-2965.03 to 5178.20)	1963.29		0.579
		VO_2_max (ml/kg/min)	145.04 (57.80 to 232.27)	42.06	0.664	0.020
		Fcon180 (Nm/kg)	1403.88 (218.02 to 2589.73)	571.81	0.336	0.022

LIR distance (m)	0.215					
		Constant	4161.81 (1792.43 to 6531.19)	1145.37		0.001
		LT2 speed (km/h)	265.75 (66.19 to 465.30)	96.46	1.000	0.011

HIR distance (m)	0.633					
		Constant	-1312.43 (-3458.05 to 833.19)	1028.98		0.217
		Fcon180 (Nm/kg)	690.07 (246.10 to 1134.05)	212.84	0.396	0.040
		F0 (N/kg)	35.59 (5.01 to 66.17)	14.66	0.222	0.025
		LT2 speed (km/h)	146.06 (14.00 to 278.12)	63.31	0.201	0.032
		Fat percentage	-25.85 (-50.47 to -1.24)	11.80	0.181	0.040

VHIR distance (m)	0.654					
		Constant	1911.33 (731.08 to 3091.60)	567.54		0.003
		Fat percentage	-19.65 (-28.21 to -11.09)	4.12	0.649	< 0.001
		Fcon180 (Nm/kg)	213.39 (42.71 to 384.08)	82.08	0.192	0.017
		30-m sprint speed (s)	-290.51 (-546.20 to -34.82)	122.95	0.159	0.028

LIR, HIR and VHIR = low- (< 13 km/h), high- (13–19 km/h) and very high-intensity (> 19 km/h) running distance during the match, Confidence interval (CI), maximal oxygen consumption (VO_2_max), speed at lactate threshold 2 (LT2 speed), concentric knee flexor peak torque with a speed of 180 degrees per second (Fcon180) and force output in loaded squat jumps (F0).

- Total distance [m] (F = 9.892, p = 0.01, SEE = 668.138) = 1106.58 + 145.04*VO_2_max [ml/kg/min] + 1403.88*Fcon180 [Nm/kg]
- LIR [m] (F = 7.589, p = 0.011, SEE = 429.890) = 4161.81 + 265.75*LT2 speed [km/h]
- HIR [m] (F = 11.360, p < 0.001, SEE = 245.274) = -1312.43 + 690.07*Fcon180 [Nm/kg] + 35.59*F0 [N/kg] + 146.06*LT2 speed [km/h] – 5.85*Fat percentage
- VHIR [m] (F = 16.141, p < 0.001, SEE = 94.898) = 1911.33 – 19.65*Fat percentage + 213.39*Fcon180 [Nm/kg] – 290.51*30-m speed [s]

Adjusted R-squared values were higher for distances of HIR and VHIR than total distance and LIR distance. The analysis shows that the most important predictors of HIR distance is Fcon180 and fat percentage for VHIR distance.

## DISCUSSION

Several statistically significant, small to large, correlations were found between match running performance and physical qualities and anthropometry in the present study. Multiple regression analysis revealed that the identified variables could predict 22–65% of match running distances. Predictions were higher for VHIR (65%) and HIR (63%) compared to LIR (22%) and total (43%) distance. The most important quality to predict total distance was VO_2_max, while the speed at the second lactate threshold best predicted LIR distance. HIR distance was influenced most by knee flexor peak concentric torque at a speed of 180 degrees per second, while body fat percentage best predicted VHIR distance.

The present study’s match running performance was lower than reported in elite players during the 2019 World Cup [1]. Especially distance covered at speeds > 19 km/h were noticeably lower (~40–65% depending on position) compared to elite players [1]. This finding is supported by previous studies, which have shown that match running ability/demands increase with competition level [2], especially in terms of HIR and sprint distance [3]. Thus, it may be surprising that there were no statistically significant differences, and mostly trivial effect sizes, in match running performance between 1^st^ team and U18-team players observed in the present study. The only statistical difference between the two teams was peak isokinetic knee extensor torque at 180 degrees per second. As there were no major differences in physical qualities between groups, this could explain why there were no observed differences in match running performance. On the other hand, running performance in a soccer match is affected by several contextual aspects [8, 9, 10] not just competition level [2, 3], physical qualities (VO_2_max [7, 19], strength [18, 20] or sprint speed [17, 18, 20]) or body composition [24]. Physical performance is suggested to be greatest in matches against similarly ranked opponents, perhaps due to a greater perceived chance of winning [8]. Based on the teams’ league ranking during the 11-week period (1^st^ team bottom three and U18-team mid-table), the U18-team played more matches against similarly ranked opponents, which could have increased match running performance compared to the 1^st^ team, reducing the otherwise expected differences.

This study’s national level players’ height and body mass were similar than previously reported in elite women players, but body fat percentage was higher compared to elite women players [14, 15, 21]. Multiple regression analyses revealed that physical qualities and anthropometry predicted 65% of VHIR distance covered in the match and body fat percentage was the most important (importance 0.649) predictor in that model. Significant negative correlations between body fat percentage and HIR (r = -0.38) and sprint running (r = -0.57) distances have been reported in professional men players previously [24]. Nevertheless, findings of the present study highlight the role of body composition also in women players. Based on the present predictive modelling, one percentage point decrease in body fat would increase VHIR distance covered in the match by 19.65 meters. Improvement in body composition would also improve body mass adjusted strength and power, consequently having a positive effect to match running performance. Thus, strength training is recommended to national level women soccer players to improve absolute strength and body mass adjusted power qualities, as well as increase lean mass, thus, improving muscle: fat ratio [36].

Other important variables to predict VHIR were Fcon180 (importance 0.192) and 30-meter sprint time (importance 0.159). Associations between VHIR and Fcon180 was expected in the present study since leg muscle thickness and architecture along with VO_2_max were predictors of high intensity running in women collegiate soccer match [19]. Also, moderate-to-strong correlations between 30-meter sprint time and HIR and sprint distances covered in the match have been previously reported [17]. Overall players performance in a 30-meter sprint test [11, 15] and in isokinetic strength tests reported in the present study were weaker than reported with elite players [37]. Thus, there appears to be a need to improve both maximum strength and power/sprint ability in the players of the present study, which should increase players’ VHIR and HIR distances in the match, based on findings of present study.

Fcon180 (importance 0.396), F0 (importance 0.222), LT2 speed (importance 0.201) and fat percentage (importance 0.181) predicted 63% of HIR distance. Thus, compared to predictors of VHIR, the role of strength qualities increases and body fat percentage decreases for this variable. Such predictors are similar to those reported by McCormack et al’s [19] regression model where VO_2_max, vastus lateralis thickness and pennation angle were the strongest predictors of HIR distance covered in women collegiate soccer matches. In the present study, knee flexor strength at relative high contraction speed and estimated body mass adjusted maximum force in ballistic movement (F0) were most important to predict HIR distance. These findings are likely in agreement because pennation angle increases alongside increases in muscle mass [38] and muscle mass is the greatest predictor of strength [39]. The only exception between studies was our model placed more emphasis on LT2 speed instead of VO_2_max to predict HIR distance, whereas VO_2_max was the only variable to describe aerobic capacity in McCormack et al. [19].

Multiple regression analyses predicted 22% of LIR and 43% of total distance covered in the match. Based on these findings, anthropometry and physical qualities have a greater influence on VHIR and HIR distance than LIR and total distance covered in the match. Mohr et al. [3] found that higher-level players outperform lower-level counterparts in HIR and sprint distance while there was no major difference in total distance covered in the match. Thus, it is possible that total and LIR distance covered in the match are more stable variables, not reliant on specific qualities or trainable characteristics. Of note, absolute VO_2_max values were lower than reported in previous studies with elite players, which averaged 50–55 ml/kg/min [7, 12, 13, 14]. Findings of the present study showed that if players’ VO_2_max would be improved to an elite level (+10 ml/kg/min) total distance covered in the match could theoretically increase by 1450 meters. The association of VO_2_max and total distance is logical, although there are varying coefficients reported between total distance and VO_2_max [7, 19]. Therefore, improving VO_2_max may have a direct impact on total distance covered during the match, but this interpretation should be confirmed in a randomized-controlled trial.

The strengths of the study were high-quality laboratory test methods (incremental treadmill test, isokinetic strength, DXA) used to measure players’ physical qualities and anthropometry. Also, the relatively high number of match observations from official league matches providing ecological validity of match demands at the national level. Further, the typically neglected use of multiple regression analysis allowed deeper insights into the relative importance of specific physical qualities and anthropometry to match running performance. This may also explain higher R² values in present study compared to previous studies, which have used simple linear regression [17, 18].

The biggest limitation of the study, as in other studies [16], was that players’ positions were not taken into consideration in analysis. Previous studies have shown that players’ running performance during the match, anthropometry and physical qualities vary between playing positions [5] and compiling all positions together would likely increase the variability in the data. In this study, subjects were national level players, so conclusions cannot be directly generalized to elite-level players in which the variance of physical qualities and anthropometry can be smaller compared to national level players [11, 12].

### Practical applications

Coaches should be aware of women players’ physical qualities and place particular emphasis on improving e.g. sprint speed, maximum (high-speed) strength, and muscle:fat ratio along with tactical and technical aspects of soccer. To improve HIR and VHIR distance in matches, the results suggest that sprint training as well as resistance and power/plyometric training should be an integral part of women players’ periodized training program. While body composition was also a predictor of HIR and VHIR, it should be noted that the body composition of young athletes is influenced by their growth and maturity status, and there is a high inter-individual variation in healthy body composition. Further, given the influence of strength variables on performance, the emphasis should be to increase muscle mass (thus reducing fat %) rather than reduce fat mass *per se*. To improve total and LIR distances, it is recommended to improve aerobic capacity through small-sided games and/or interval training.

## CONCLUSIONS

Physical qualities and anthropometry are related to, and can predict 22–65% of, national level women players’ running performance during matches. Physical qualities and anthropometry demonstrated greater prediction magnitudes of very high-intensity running (> 19 km/h) (65%) and high-intensity running (13–19 km/h) (63%) than to low-intensity (< 13 km/h) (22%) and total (43%) running distances. To increase total distance covered in the match, the most important quality to improve would be VO_2_max. To increase high-intensity and very high-intensity running distances, improvement of high-speed (especially knee flexor) maximum force production, body composition, and sprint speed are identified as the most important in the present study.

## Conflict of interest declaration

The authors declare no conflict of interest.
